# Hamman’s syndrome triggered by the onset of type 1 diabetes mellitus accompanied by diabetic ketoacidosis

**DOI:** 10.1007/s00592-016-0871-z

**Published:** 2016-06-13

**Authors:** Shinji Kamei, Hideaki Kaneto, Akihito Tanabe, Ryo Shigemoto, Shintaro Irie, Yurie Hirata, Maiko Takai, Kenji Kohara, Masashi Shimoda, Tomoatsu Mune, Kohei Kaku

**Affiliations:** Department of Diabetes, Metabolism and Endocrinology, Kawasaki Medical School, 577 Matsushima, Kurashiki, 701-0192 Japan

Dear Editor,

Hamman’s syndrome, which is also known as spontaneous pneumomediastinum, could be induced in various situations such as labor and delivery [1]. The potential mechanism for Hamman’s syndrome is as follows: The vulnerability of alveolar walls by some reason increases air pressure, which leads to alveolar rupture. Then, spilled air peels the connective tissue and finally enters into mediastinal space through pulmonary hilum. Hamman’s syndrome is one of the relatively rare pulmonary diseases, and there are few reports showing the case of Hamman’s syndrome triggered by the onset of type 1 diabetes mellitus accompanied by diabetic ketoacidosis. However, recently we experienced three cases of young male subjects with Hamman’s syndrome triggered by the onset of type 1 diabetes mellitus accompanied by diabetic ketoacidosis. These cases are very rare, and there were many similarities among these three cases.

Clinical characteristics of these three cases were as follows: age (years old): 29, 20, 20 (case 1, case 2, case 3); gender: male, male, male; HbA1c (%): 12.0, 12.3, 6.9; plasma glucose level (mg/dL): 1298, 1543, 783; pH: 6.89, 7.10, 7.24; total ketone body concentration in blood (µmol/L): 1662, 20,960, 15,500; acetoacetic acid (µmol/L): 734, 5080, 2960; 3-hydroxybutyric acid (µmol/L): 928, 15,880, 12,540; WBC (/mL): 37,370, 27,590, 7050; CRP (mg/dL): 4.01, <0.03, 0.76, respectively. Type of diabetes in all three subjects was type 1A, and the autoantibodies of the pancreas were positive: anti-GAD antibody (U/mL): 157.7, 3.0, 1.5 (case 1, case 2, case 3); anti-IA-2 antibody (U/mL): 2.6, 5.6, <0.4, respectively. Marked reduction in body weight was observed: BMI on admission (kg/m^2^): 25.1, 13.7, 19.0; BMI before the onset of type 1 diabetes (kg/m^2^): 29.3, 17.2, 21.7; recent weight loss (kg/duration): 10 kg/1 month, 11 kg/2 weeks, 8 kg/1 week, respectively. All three cases experienced vomiting very frequently, and case 1 and 2, but not case 3, experienced chest pain. On physical examination, subcutaneous emphysema was observed in all three cases. In chest X-ray, pneumomediastinum was observed in these three cases (red arrows) (Fig. 1). Also, in computed tomography, such pneumomediastinum was confirmed in these three cases (red circles) (Fig. 1). In case 1, pneumonia image was observed in lower right lung field (blue arrows and blue circle). In addition, subcutaneous emphysema was observed in all cases.

**Fig. 1:**
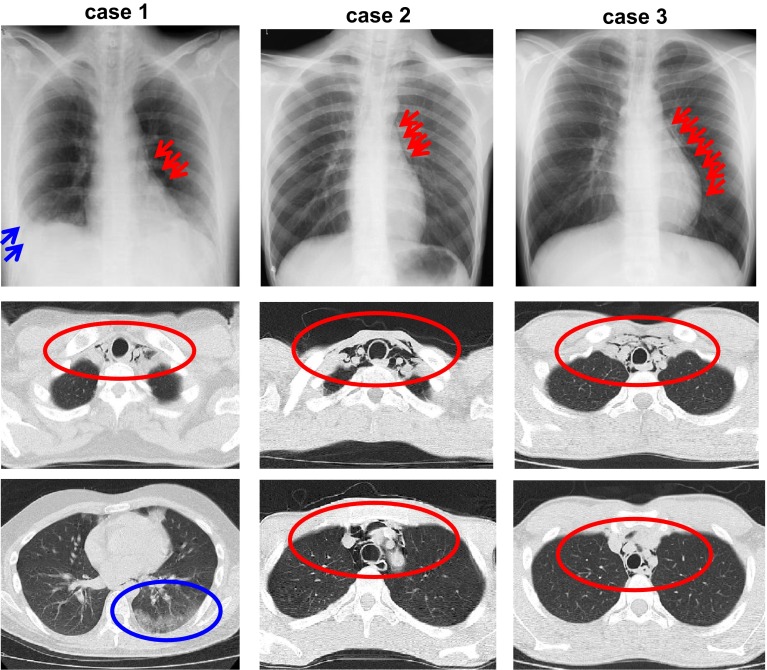
Chest X-ray (*upper panels*) and computed tomography (*middle* and *lower panels*) in three cases with Hamman’s syndrome triggered by the onset of type 1 diabetes mellitus accompanied by diabetic ketoacidosis. Pneumomediastinum is observed in these three cases (*red arrows* and *red circles*). In case 1, pneumonia image is observed in lower right lung field (*blue arrows* and *blue circle*)

We immediately started antibiotics therapy in order to prevent the progression of inflammation. Mediastinal emphysema image in chest X-ray disappeared in several days after starting the therapy. The number of days required for the disappearance of emphysema image in these three cases was 4, 9 and 5 days, respectively. The number of days until the complete recovery was 15, 9 and 6 days, respectively. There were no sequelae and no recurrence of emphysema at all in all cases.

It is thought that in cases with diabetic ketoacidosis air pressure could be increased by Kussmaul’s breathing or repeated vomiting [2, 3]. Therefore, we think that repeated vomiting might have triggered the onset of Hamman’s syndrome in these three subjects. In addition, decrease in pulmonary surfactant observed with diabetic ketone acidosis could lead to the vulnerability of alveolar walls [4]. Therefore, although speculative, such phenomena might have been also involved in the onset of Hamman’s syndrome in these cases.

In conclusion, we should be aware of the possibility that Hamman’s syndrome is induced by the onset of type 1 diabetes mellitus accompanied by diabetic ketoacidosis. Since such pneumomediastinum sometimes becomes very severe and leads to a fatal outcome [5], we think it would be better to perform computed tomography for many subjects with diabetic ketoacidosis.

## References

[1] Zapardiel I, Delafuente-Valero J, Diaz-Miguel V, Godoy-Tundidor V, Bajo-Arenas JM (2009). Pneumomediastinum during the fourth stage of labor. Gynecol Obstet Invest.

[2] Bullaboy CA, Jennings RB, Johnson DH, Coulson JD, Young LW, Wood BP (1989). Radiological case of the month. Pneumomediastinum and subcutaneous emphysema caused by diabetic hyperpnea. Am J Dis Child.

[3] Pooyan P, Puruckherr M, Summers JA, Byrd RP, Roy TM (2004). Pneumomediastinum, pneumopericardium, and epidural pneumatosis in DKA. J Diabetes Complicat.

[4] Sahebjami H, Vassallo CL, Wirman JA (1978). Lung mechanics and ultrastructure in prolonged starvation. Am Rev Resp Dis.

[5] Kaneki T, Kubo K, Kawashima A, Koizumi T, Sekiguchi M, Sone S (2000). Spontaneous pneumomediastinum in 33 patients: yield of chest computed tomography for the diagnosis of the mild type. Respiration.

